# Immune Activation and CD8^+^ T-Cell Differentiation towards Senescence in HIV-1 Infection

**DOI:** 10.1371/journal.pbio.0020020

**Published:** 2004-02-17

**Authors:** Laura Papagno, Celsa A Spina, Arnaud Marchant, Mariolina Salio, Nathalie Rufer, Susan Little, Tao Dong, Gillian Chesney, Anele Waters, Philippa Easterbrook, P. Rod Dunbar, Dawn Shepherd, Vincenzo Cerundolo, Vincent Emery, Paul Griffiths, Christopher Conlon, Andrew J McMichael, Douglas D Richman, Sarah L Rowland-Jones, Victor Appay

**Affiliations:** 1Medical Research Council Human Immunology Unit, Institute of Molecular MedicineJohn Radcliffe Hospital, OxfordUnited Kingdom; **2**Institute of Infectious and Tropical Diseases, University of MilanL. Sacco Hospital, MilanItaly; **3**San Diego Veterans Affairs Research Center for AIDS and HIV Infection, University of CaliforniaSan Diego, La JollaCalifornia; **4**National Center of Competence in Research Molecular Oncology, Swiss Institute for Experimental Cancer ResearchEpalingesSwitzerland; **5**Department of HIV/GUM, The Guy'sKings', and St Thomas' School of Medicine, LondonUnited Kingdom; **6**Department of Virology, Royal Free and University College Medical SchoolLondonUnited Kingdom; **7**Nuffield Department of Medicine, John Radcliffe HospitalOxfordUnited Kingdom

## Abstract

Progress in the fight against the HIV/AIDS epidemic is hindered by our failure to elucidate the precise reasons for the onset of immunodeficiency in HIV-1 infection. Increasing evidence suggests that elevated immune activation is associated with poor outcome in HIV-1 pathogenesis. However, the basis of this association remains unclear. Through ex vivo analysis of virus-specific CD8^+^ T-cells and the use of an in vitro model of naïve CD8^+^ T-cell priming, we show that the activation level and the differentiation state of T-cells are closely related. Acute HIV-1 infection induces massive activation of CD8^+^ T-cells, affecting many cell populations, not only those specific for HIV-1, which results in further differentiation of these cells. HIV disease progression correlates with increased proportions of highly differentiated CD8^+^ T-cells, which exhibit characteristics of replicative senescence and probably indicate a decline in T-cell competence of the infected person. The differentiation of CD8^+^ and CD4^+^ T-cells towards a state of replicative senescence is a natural process. It can be driven by excessive levels of immune stimulation. This may be part of the mechanism through which HIV-1-mediated immune activation exhausts the capacity of the immune system.

## Abstract 2

HIV-1 infection induces activation and differentiation of CD8^+^ T-cells, resulting in replicative senescence. This may be part of the mechanism through which HIV-1 exhausts the capacity of the immune system

## Introduction

During primary human immunodeficiency virus 1 (HIV-1) infection, the immune system appears to respond appropriately in order to prevent viral spread, with the mounting of a strong HIV-specific CD8^+^ T-cell response and a corresponding reduction in viraemia (Koup et al. 1994). In common with the majority of persistent viruses, HIV has developed a number of strategies to evade host immunity (Alcami and Koszinowski 2000). Continuous adaptive mutation (Borrow et al. 1997) and destruction or impairment of elements necessary for an optimal immune response (e.g., CD4^+^ T-cells and antigen-presenting cells) (Kalams and Walker 1998) may explain the failure of antiviral immunity to eradicate the virus. However, unlike most other persistent viruses, HIV-1 progressively destroys the immune system, resulting in acquired immunodeficiency syndrome (AIDS) and death. The precise mechanisms by which immune function is lost remain the subject of considerable controversy. In addition to elevated T-cell turnover and an increase in the proportion of highly differentiated antigen-experienced CD8^+^ and CD4^+^ T-cells during HIV infection (Wolthers et al. 1996b; Appay et al. 2002c), HIV-infected individuals are characterised by decreased thymic output (Douek et al. 1998) and reduced naïve T-cell numbers (Roederer et al. 1995; Hellerstein et al. 1999, Hellerstein et al. 2003), which reflect a diminished capacity to renew the pool of T-cells.

Increasing evidence suggests an association between high levels of immune activation and poor outcome in HIV-infected individuals (Giorgi et al. 1993; Hazenberg et al. 2000a, Hazenberg et al. 2003; Grossman et al. 2002; Sousa et al. 2002), although the underlying mechanism remains unclear. This is supported by studies of sooty mangabeys and African green monkeys, the natural hosts of simian immunodeficiency virus (SIV), which survive SIV infection and are characterised by low immune activation, in striking contrast to rhesus macaques, for which SIV infection is fatal (Kaur et al. 1998; Broussard et al. 2001; Silvestri et al. 2003). To gain further insights into the mechanisms involved, we have studied the potential interplay among immune activation, CD8^+^ T-cell differentiation, and outcome in the context of HIV-1 pathogenesis. We report here that T-cell activation and differentiation are closely related, and that HIV-1 induces immune activation directly and indirectly, which results in differentiation of CD8^+^ T-cells towards replicative senescence.

## Results

### HIV-Infected Subjects

Our study involved the analysis of two distinct groups of HIV-1-infected individuals. On one hand, we performed a longitudinal analysis of T-cell subsets during acute HIV-1 infection and its resolution. To examine the effect on T-cells of elevated immune activation associated with an episode of vigourous HIV replication (particularly evident at time of high HIV-1 viraemia, such as the acute infection phase), T-cells were studied in individuals during HIV acute infection and later on—postacute—when viral replication was suppressed following the start of antiretroviral therapy (ART) (Table 1). These donors were diagnosed at an early stage of primary infection: before or at the time of HIV-1 seroconversion. On the other hand, we carried out a cross-sectional study of HIV-infected untreated individuals at different stages of infection, to draw a correlation between their T-cell characteristics and clinical status. For this purpose, untreated HIV-infected donors were classified into three different groups: acute infection, chronic infection with no sign of progression (infected for more than 10 y with a CD4^+^ count above 500 per milliliter and mean viral load of 10^4^ copies/ml), and chronic infection with signs of disease progression (with decreasing CD4^+^ count, 500 < *x* < 130 per milliliter, and mean viral load of 7 × 10^4^ copies/ml). In addition to analysing whole CD8^+^ T-cell populations in these individuals, we have used a panel of tetramers to study the phenotypic evolution of CD8^+^ T-cells specific for HIV, cytomegalovirus (CMV), Epstein–Barr virus (EBV), and influenza. Although this approach focuses on a limited number of viral epitopes (restricted by the number of tetramers available), it remains the only way to avoid stimulation of the cells in order to detect them (e.g., by interferon-γ [IFN-γ] secretion), which may alter cellular phenotype and does not enable the detection of all cells.

**Table 1:**
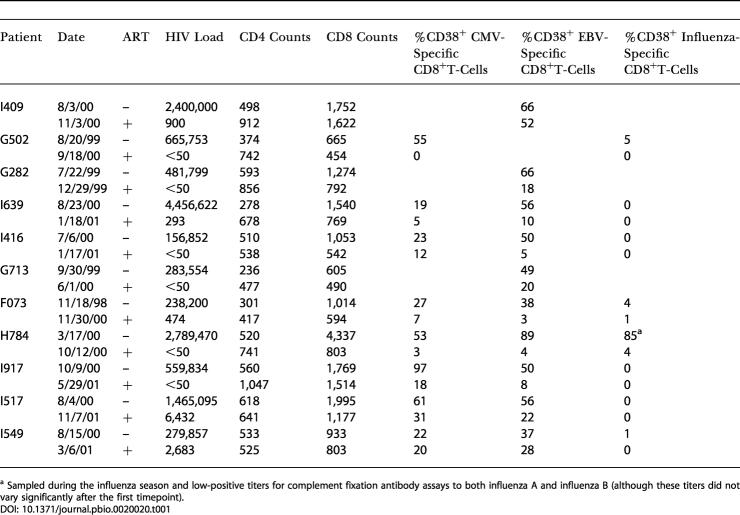
Clinical Characteristics and Percentages of Activated HIV-Nonspecific CD8^+^ T-Cells in Donors Studied during Both Acute and Postacute HIV-1 Infection Stages

^a^ Sampled during the influenza season and low-positive titers for complement fixation antibody assays to both influenza A and influenza B (although these titers did not vary significantly after the first timepoint)

### Direct and Indirect T-Cell Activation during Acute HIV-1 Infection

**Figure 1:**
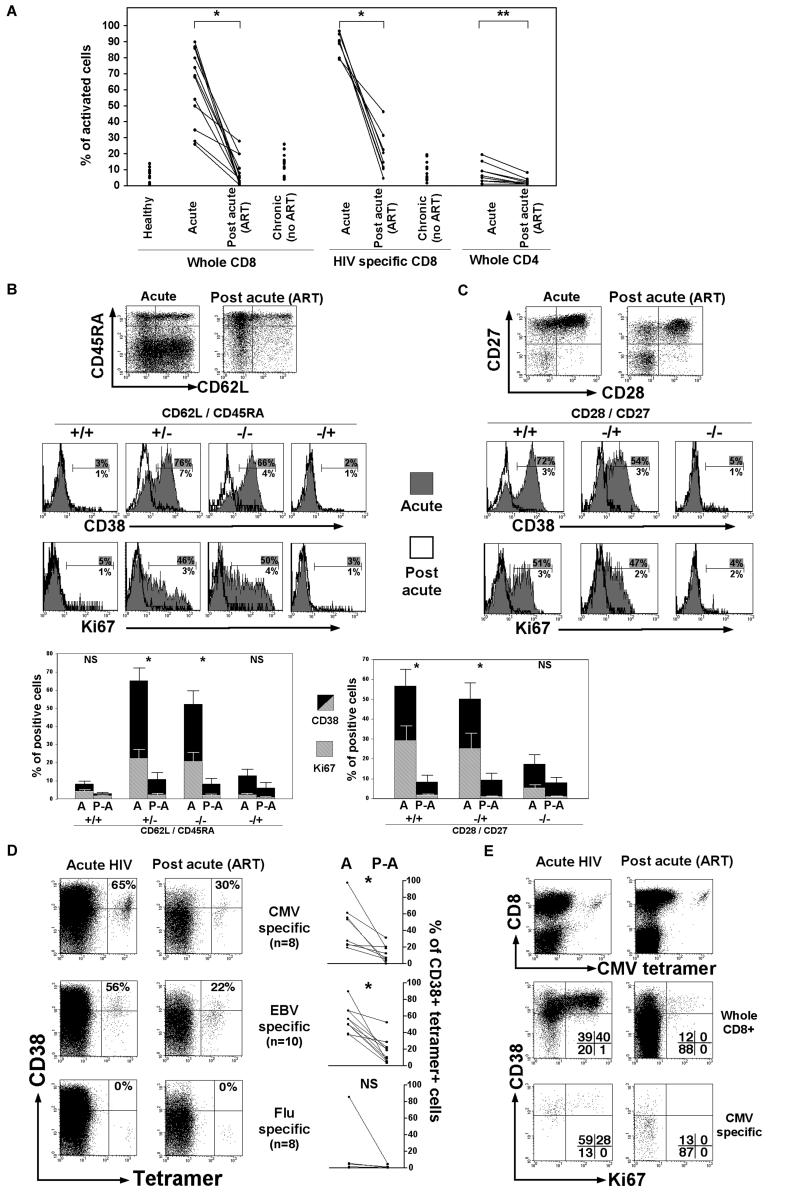
CD8^+^ T-Cell Activation during Acute HIV-1 Infection (A) Percentages of activated CD38^+^ cells (gated on whole CD8^+^ T-cells, HIV tetramer-positive CD8^+^ T-cells, or whole CD4^+^ T-cells) in donors during acute HIV-1 infection and later postacute on ART (*n* = 12); healthy donors (*n* = 11) and untreated donors with nonprogressing chronic infection (*n* = 12) are also shown. (B and C) CD38 and Ki67 expression on CD8^+^ T-cell subsets defined by CD45RA/CD62L (B) or CD28/CD27 (C) expression, shown in one single donor from acute to postacute (on ART) HIV-1 infection. Percentages of positive cells are shown. Means (± SEM) of CD38^+^ and Ki67^+^ CD8^+^ T-cells for ten patients are also shown; statistics concern CD38 expression. (D) Staining for the activation marker CD38 on CMV-, EBV-, or influenza A virus-specific CD8^+^ T-cells during acute and postacute (on ART) HIV-1 infection in a single donor. Percentages of CD38^+^ tetramer-positive CD8^+^ T-cells are shown. Data on all donors (see Table 1) are also shown. (E) Activation (CD38 and Ki67 staining) of CMV-specific CD8^+^ T-cells or whole CD8^+^ T-cell population during acute and postacute (on ART) HIV-1 infection in a single donor. Percentages of cells present in quadrants are shown. Statistics: * *p* < 0.002, ** *p* < 0.01, NS = nonsignificant, with the nonparametric Mann–Whitney test.

CD38 was used as a marker of activation; cells expressing high levels of CD38 (Appay et al. 2002b) were considered as being activated. During acute HIV-1 infection, HIV-specific CD8^+^ T-cells were strongly activated, and, intriguingly, activation of the CD8^+^ T-cell compartment as a whole was particularly high, reaching to levels of 80%–90%, in contrast to CD4^+^ T-cells, which show much less activation (Figure 1A). In order to shed light on the elevated level of activation experienced by the CD8^+^ T-cell population, we examined which CD8^+^ T-cell subsets were activated and whether all activated cells were HIV-specific. Naïve cells exhibited a slight increase in Ki67 (proliferation marker) expression during acute infection (*p* = 0.03), in keeping with activation-related proliferation of this subset, as previously described (Hazenberg et al. 2000b). However, little or no difference in activation levels CD38^+^ between acute and postacute infection stages was observed within the naïve CD8^+^ T-cell subset (CD62L^+^/CD45RA^+^) and antigen-experienced CD45RA^+^ (quiescent [Dunne et al. 2002; van Leeuwen et al. 2002]) CD8^+^ T-cells, in contrast to the rest of antigen-experienced CD8^+^ T-cells (Figure 1B). This indicates that most activated CD8^+^ T-cells are or have become antigen-experienced. According to the expression of the costimulatory receptors CD28 and CD27, antigen-experienced CD8^+^ T-cells can be positioned along a putative linear model of differentiation or post-thymic development: early (CD28^+^/CD27^+^), intermediate (CD28^−^/CD27^+^), and late (CD28^−^/CD27^−^) differentiated subsets (Appay et al. 2002a). While both CD28^+^/CD27^+^ and CD28^−^/CD27^+^ T-cell subsets expressed high levels of CD38 and Ki67 during acute infection, CD28^−^/CD27^−^ T-cells exhibited little activation and proliferation despite increased proportions of these cells following acute infection (Figure 1C), suggesting the differentiation into this subset of earlier differentiated cells following activation.

Surprisingly, from the analysis of CD8^+^ T-cells specific for non-HIV viral antigens in donors with suitable human leukocyte antigen (HLA) type (HLA-A*0201 for CMV, EBV, and influenza A virus; HLA-B*0701 for CMV; and HLA-B*0801 for EBV), both CMV- and EBV-specific CD8^+^ T-cells displayed significant levels of activation exclusively during acute HIV infection, compared to chronic infection (*p* < 0.002) (Figure 1D; see Table 1). Activated cells specific for non-HIV viral antigens also participated in the expansion of the CD8^+^ T-cell population observed in HIV primary infection, as shown by expression of the proliferation marker Ki67 (Figure 1E). Plasma DNA levels of CMV and EBV in these study subjects were below detection limits of the assays and thus did not provide evidence of high levels (greater than 400 genomes per milliliter) of systemic reactivation (data not shown). However, the observation of nonactivated influenza A virus-specific CD8^+^ T-cells (Figure 1D), in contrast to CMV- or EBV-specific CD8^+^ T-cells (*p* < 0.01), strongly suggests that the stimulation of these cells associated with HIV-1 infection is due to reactivation of pathogens such as CMV and EBV, rather than as a result of bystander activation. Overall, these data show that HIV-1 infection leads to activation of antigen-experienced CD8^+^ T-cells at early stages of differentiation, both in direct (HIV-specific) and indirect (HIV-nonspecific) manners.

### Activation-Induced T-Cell Differentiation 

**Figure 2:**
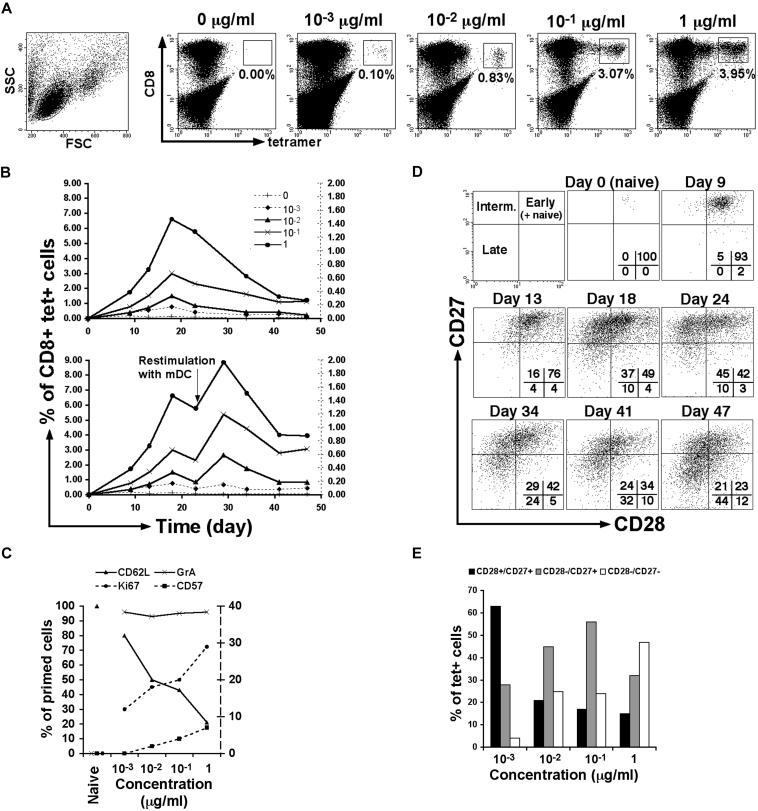
In Vitro Priming of Antigen-Specific CD8^+^ T-Cells (A) Representative stainings for melan-A-specific CD8^+^ T-cells following priming of naïve cells from healthy donor PBMCs by autologous mature DCs loaded with various concentrations of antigen. Cells are gated on lymphocytes 47 d after priming. Percentages of melan-A tetramer-positive CD8^+^ T-cells are shown. (B) Percentages of melan-A-specific CD8^+^ T-cells over time following priming at day 0 with mature DCs loaded with various concentrations of antigen, with no restimulation or with restimulation using mature DCs at day 25. The legend indicates the concentration of melan-A–peptide used in microgram per milliliter; populations generated with 0 or 10^−3^ μg/ml of antigen are plotted on the right-hand side Y axis. (C) Percentages of melan-A tetramer-positive CD8^+^ T-cells expressing granzyme A, Ki67, CD62L, or CD57 according to antigen concentration used, at day 30 following priming. Ki67 and CD57 expressions are plotted on the right-hand side Y axis. (D) CD28 and CD27 expression on melan-A tetramer-positive CD8^+^ T-cells in PBMC (day 0), and over time following priming with 1 μg/ml of antigen. Percentages of cells present in quadrants are shown. The model of CD8^+^ T-cell differentiation based on CD28 and CD27 expression is illustrated (top left panel). (E) Distribution of the melan-A-specific CD8^+^ T-cells into the distinct differentiated subsets according to antigen concentration used, at day 47 following priming. Similar observations were made whether the cells were subjected to a second round of stimulation or not. Data are representative of three independent experiments.

The potential relationship between T-cell activation and differentiation was first studied using a system of in vitro priming of naïve CD8^+^ T-cells by dendritic cells (DCs), which represents a useful model to analyse the generation of antigen-experienced CD8^+^ T-cells. This system is based on the existence in normal human donors of a significant number of naive CD8^+^ T-cells (reactive for the HLA-A2-restricted melan-A antigen [Dutoit et al. 2002; Zippelius et al. 2002]), which can be primed by autologous matured DCs loaded with specific peptides to become antigen-experienced cells (Salio et al. 2001). Although we cannot with certainty extend our interpretation of data from this assay system beyond the in vitro conditions (i.e., signals involved in T-cell differentiation, apoptosis, or both, as well as homeostatic signals, may be absent or differ from the in vivo situation), this system represents a unique opportunity to study the priming of naïve CD8^+^ T-cells using human material. We used a range of concentrations of the melan-A antigen loaded onto professional antigen-presenting cells to generate different levels of stimulation. Mature DCs do not persist very long in culture (2–3 d); moreover, the half-life of class I MHC–peptide complexes on mature DCs is rather short (Cella et al. 1999); therefore, the results reflect increasing antigen doses from a single round of antigen exposure. We observed a close relationship between the level of stimulation induced and the size of the resulting antigen-specific CD8^+^ T-cell population (Figure 2A). This relationship was steady, as maintained over time, following priming of naïve cells and following a second round of stimulation of the antigen-experienced cells with antigen-loaded matured DCs (Figure 2B). The priming of naïve cells (granzyme A-negative) was successfully initiated at all antigen concentrations, as shown by the expression of the cytotoxic factor granzyme A in all melan-A-specific CD8^+^ T cells (Figure 2C). Increasing concentrations of antigen were associated with increasing activation levels and proliferation, indicated by increased expression of Ki67 and declining expression of CD62L (Figure 2C). The analysis of the differentiation phenotype (based on CD28 and CD27 expression) throughout the priming of the cells provided in vitro confirmation of the hypothetical model of CD8^+^ T-cell differentiation observed ex vivo (Hamann et al. 1999; Appay et al. 2002a): starting from a population with naïve characteristics (CD28^+^/CD27^+^/CD62L^+^/CD45RA^+^/granzyme A^−^) at day 0 (data not shown), antigen-primed cells lost sequentially expression of CD28 and CD27 (Figure 2D). Following priming, the differentiation phenotype of the melan-A-specific CD8^+^ T-cells varied according to the level of stimulation induced, with high antigen load resulting in further differentiation of the cells (Figure 2E). These data show that there is a close correlation among the level of activation, size, and differentiation of the antigen-specific CD8^+^ T-cells.

**Figure 3:**
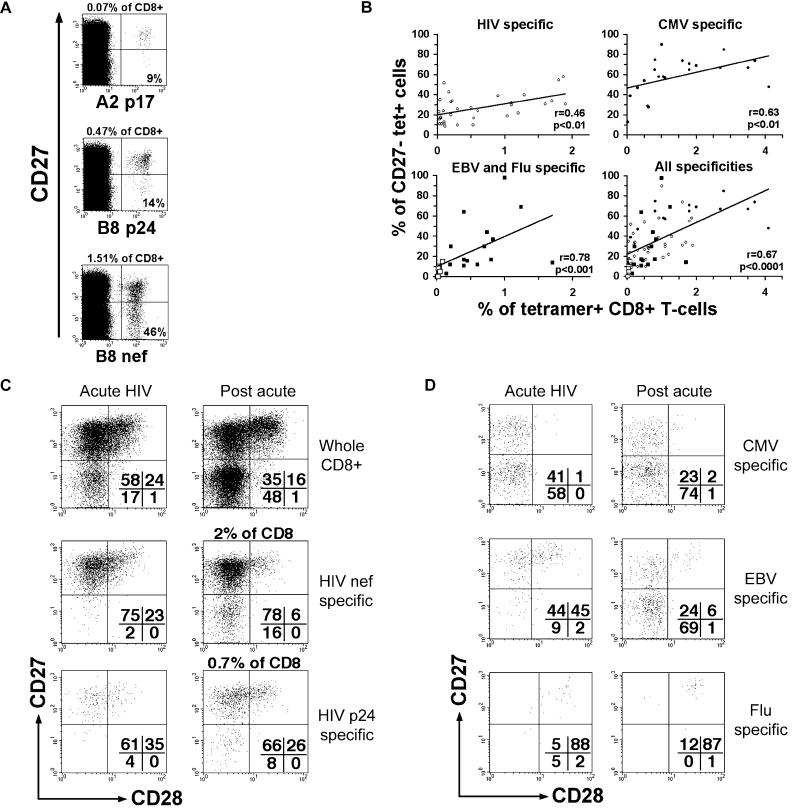
Activation and Differentiation of Antigen-Specific CD8^+^ T-Cells during HIV-1 Infection (A) Representative staining for the differentiation marker CD27 on three HIV-specific (HLA-B8 nef, HLA-A2 p17, and HLA-B8 p24) populations in a single HIV-1-infected donor. Numbers show percentages of tetramer-positive CD8^+^ T-cells (outside the quadrants) and percentages of CD27^−^ tetramer-positive cells (inside the quadrants). (B) Correlation between size (percentage of tetramer-positive CD8^+^ T-cells) and differentiation (percentages of CD27^−^ tetramer-positive cells) of CD8^+^ T-cells specific for HIV antigens (including HLA-A2 p17, pol, HLA-B7 nef, gp41, HLA-B8 nef, p24, and HLA-B57 p24) (open circles), CMV antigens (including HLA-A2, B7, and B35 pp65) (filled circles), EBV (HLA-A2 BMLF1, HLA-B8 BZLF1, EBNA3A) (filled squares), and influenza (HLA-A2 matrix) (open squares) antigens or all antigens together. These populations were studied in individuals with chronic infection for HIV, CMV, or EBV (independently from clinical status). *P* values were obtained using the nonparametric Spearman rank correlation test. (C) CD28 and CD27 expression on whole, HIV nef-, or p24-specific CD8^+^ T-cells during acute and postacute (on ART) HIV-1 infection in a single donor. (D) CD28 and CD27 expression on CMV-, EBV-, or influenza-specific CD8^+^ T-cells during acute and postacute (on ART) HIV-1 infection in a single donor. Percentages of cells present in quadrants are shown.

**Figure 4:**
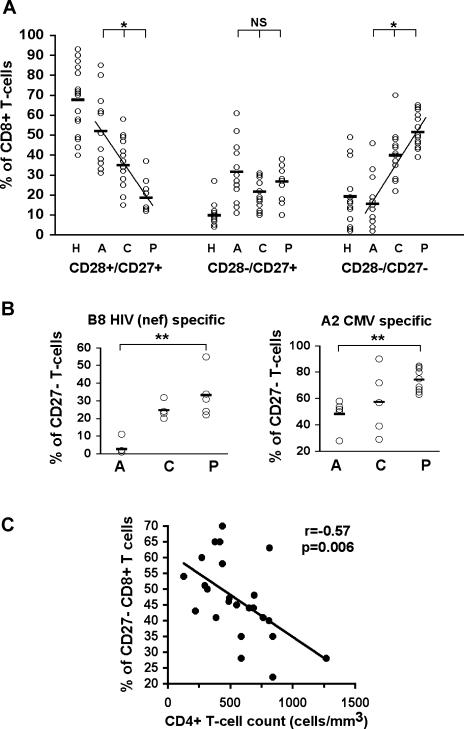
CD8^+^ T-Cell Differentiation and HIV-1 Disease Progression (A) Distribution of the CD8^+^ T-cell population in differentiated subsets (CD28^+^/CD27^+^ early, CD28^−^/CD27^+^ intermediate, and CD28^−^/CD27^−^ late) through the course of HIV-1 infection. Abbreviations: H, healthy (*n* = 15); A, acute HIV infection (*n* = 11); C, chronic HIV infection nonprogressor (no ART; *n* = 14); P, chronic HIV infection with signs of disease progression (no ART; *n* = 10). Statistics: * *p* < 0.0001 with the ANOVA test and *p* < 0.005 between each group. (B) Percentages of CD27^−^ CD8^+^ T-cells that are specific for HLA-B8 HIV (nef) or HLA-A2 CMV in HIV-1-infected individuals at different stages of infection. Statistics: ** *p* < 0.005 with the nonparametric Mann–Whitney test. (C) Inverse correlation between CD4^+^ T-cell counts and percentage of highly differentiated CD27^−^ cells in the whole CD8^+^ T-cell population of HIV-1-infected donors during chronic infection (untreated nonprogressors and progressors). The *p* value was obtained using the nonparametric Spearman rank correlation test.

This relationship was confirmed by ex vivo analysis of antigen-experienced CD8^+^ T-cells. Despite that the majority of HIV-specific CD8^+^ T-cells are usually found at an intermediate stage of differentiation (Appay et al. 2002a), certain of these populations exhibit a significant percentage of late-differentiated CD8^+^ T-cells, as exemplified by the analysis of three HIV-1-specific CD8^+^ T-cell populations in a single individual (Figure 3A). The examination of the differentiation state (percentage of CD27^−^ in the tetramer-positive cells) and the size (percentage of tetramer-positive cells in the whole CD8 population) of a variety of HIV-specific CD8^+^ T-cell populations in several donors revealed a correlation between these two parameters (Figure 3B). A similar correlation was also found in the case of CMV-specific populations (although these cells are usually more differentiated, as previously described [Appay et al. 2002a]), as well as in EBV- and influenza-specific CD8^+^ T-cells. The correlation between differentiation and population size becomes highly significant when data on all specificities are combined. Following acute HIV infection and related strong activation, HIV-specific CD8^+^ T-cells displayed increased percentages of CD28^−^/CD27^−^ cells (especially with larger populations) (Figure 3C; Figure 4B). The differentiation phenotype of non-HIV-specific CD8^+^ T-cells could also vary from acute to postacute HIV infection stages in relation to activation: while the differentiation phenotype of influenza A virus-specific cells remained unchanged, CMV- and (although less frequently) EBV-specific CD8^+^ T-cells became further differentiated (Figure 3D; Figure 4B). This is in keeping with a recent report, which shows increased differentiation of EBV-specific CD8^+^ T-cells during HIV-1 infection (van Baarle et al. 2002a). Taken together, these data indicate that the immune activation induced in the context of HIV-1 infection can result in the differentiation of T-cells specific for HIV-1 as well as other pathogens such as CMV and EBV, which may explain the increase in the proportions of highly differentiated cells observed during HIV-1 infection.

### Increased T-Cell Differentiation with Progression to AIDS

Persistent and continuous replication is a hallmark of HIV-1 infection, along with chronic activation and constant turnover of T-cells, and these factors are now thought of as playing a critical role in HIV pathogenesis and disease progression (Giorgi et al. 1993; Hazenberg et al. 2000a; Grossman et al. 2002; Hellerstein et al. 2003). The detailed distribution of the CD8^+^ T-cell population along the pathway of differentiation during HIV-1 infection was analysed in a cross-sectional study of individuals at different stages of infection. It revealed an increase in the proportion of highly differentiated CD8^+^ T-cells associated with HIV disease progression (Figure 4A). Increased proportions of CD28^−^/CD27^+^ CD8^+^ T-cells during acute HIV-1 infection are likely to reflect expansion of HIV-specific CD8^+^ T-cells. The enrichment in highly differentiated CD8^+^ T-cells from acute infection onwards included virus-specific cells, as exemplified by the analysis of populations specific for one HIV epitope or one CMV epitope (Figure 4B). The study of individuals during chronic infection (including nonprogressors and donors with evidence of disease progression, both untreated) revealed an inverse correlation between the overall percentage of highly differentiated cells and CD4^+^ T-cell count, as an indicator of disease progression (Figure 4C). No significant correlation emerged between the differentiation state of virus-specific CD8^+^ T-cell populations and CD4^+^ T-cell count; a larger number of virus-specific CD8^+^ T-cell populations studied may be required. A problem with the interpretation of increased numbers of highly differentiated T-cells relates to the controversy around the significance of these cells. Some investigators regard these cells as the effector-type population, conferring optimum protective immunity (van Baarle et al. 2002b; Zhang et al. 2003), but for others, these cells have lost their capacity to proliferate and their incidence may reflect ageing of the lymphocyte population (Effros et al. 1996; Globerson and Effros 2000; Appay and Rowland-Jones 2002b).

### Replicative Senescence and Increased T-Cell Differentiation

**Figure 5:**
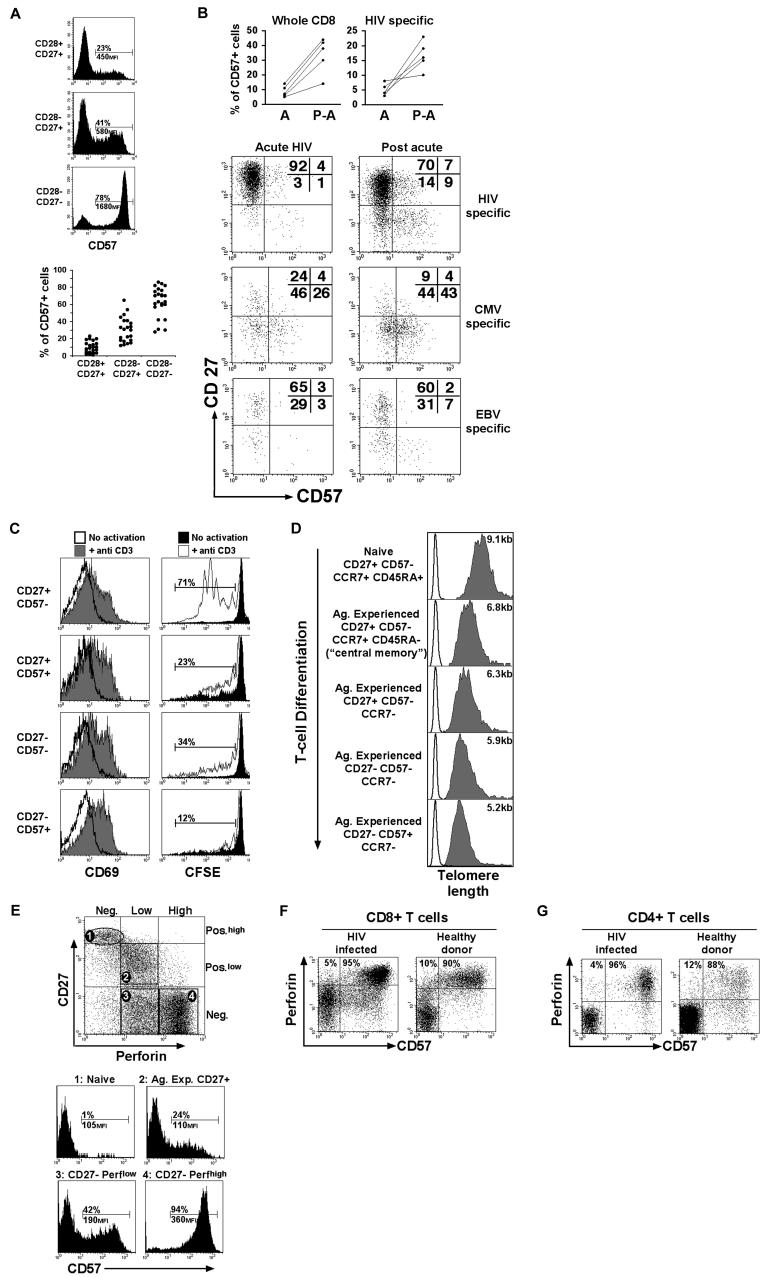
CD8^+^ T-Cell Differentiation and Senescence (A) Expression of the replicative senescence-associated marker CD57 on antigen-experienced CD8^+^ T-cell subsets. The percentage and mean fluorescence intensity for the CD57^+^ cells are shown for one single donor. Data on several donors (HIV-1-infected or healthy) are also shown (*n* = 24). (B) Expression of CD57 on CD8^+^ T-cells (whole population or antigen-specific) from acute to postacute (on ART) HIV-1 infection. (C) CD69 expression and CFSE proliferation profile for CD8^+^ T-cell subsets gated on the basis of CD57 and CD27 expression following stimulation with anti-CD3 antibodies. PBMCs were analysed for CD69 expression after 18 h and CFSE labeling after 6 d. Percentages of proliferating cells (with background subtracted) are indicated. Representative results from three experiments (one HIV-infected and two healthy donors) are shown. (D) Telomere length measurement by flow FISH on naïve and antigen-experienced CD8^+^ T-cell subsets FACS-sorted on the basis of CD57, CD27, CCR7, and CD45RA expression. The average length of telomeres was obtained by substracting the mean fluorescence of the background control (no probe; open histogram) from the mean fluorescence obtained from cells hybridised with the FITC-labeled telomere probe (gray histogram). Representative results from two experiments (on healthy donors) are shown. (E) CD57 and perforin expression in the CD8^+^ T-cell population dissected into naïve (CD27^+high^, perforin-negative), antigen-experienced CD27^+^ (perforin^low^), and antigen-experienced CD27^−^ perforin^low^ or perforin^high^ subsets. The percentage and mean fluorescence intensity for the CD57^+^ cells are indicated. (F) Representative staining for perforin and CD57 in CD8^+^ T-cells from a HIV-1-infected or a healthy donor. Percentages of cells present in the top quadrants are shown. (G) Representative staining for perforin and CD57 in CD4^+^ T-cells from an HIV-1-infected or a healthy donor. Percentages of cells present in the top quadrants are shown.

As CD8^+^ T-cells differentiate further, they express increasing levels of CD57 (Figure 5A), a marker that has recently been associated with a state of replicative senescence (Brenchley et al. 2003). This is in line with the observation of increased CD57 expression on CD8^+^ T cells following acute HIV infection, including cells specific for HIV, as well as other specificities, such as CMV- and EBV-specific cells (Figure 5B). Increased CD57 expression in association with further T-cell differentiation was also seen following priming of T-cells in vitro (see Figure 2C), although this remained relatively modest (below 10%), possibly due to the high susceptibility to activation induced cells death of CD57^+^ T-cells (Brenchley et al. 2003; unpublished data) in the interleukin-2 (IL-2)-supplemented assay conditions. In keeping with the finding by Brenchley et al. (2003), we observed that highly differentiated CD27^−^/CD57^+^ CD8^+^ T-cells exhibited a reduced capacity to proliferate despite being activated following stimulation with anti-CD3 antibodies (−/+ addition of IL-2) (Figure 5C). In addition, we measured telomere length in CD8^+^ T-cell subsets at different stages of differentiation. The telomere length reflects the mitotic history of cells: in lymphocytes, every cell division shortens the telomeres by approximately 30–60 basespairs (Rufer et al. 1998), until the cells lose their capacity to proliferate any longer. The induction of human telomerase expression (necessary for the maintenance of telomere length) has recently been shown to decrease in T-cells that have expanded in vivo upon antigen encounter (Roth et al. 2003). Shortening of the telomeres appears to occur progressively along T-cell differentiation (Figure 5D) so that highly differentiated CD27^−^/CD57^+^ cells display the shortest telomeres, with lengths (4–5 kb) equivalent to those observed in antigen-experienced CD8^+^ T-cells from the elderly (Rufer et al. 1999). All together, these data support the view that T-cells exhibit increasing characteristics of replicative senescence as they differentiate further. The assumption that CD28^−^/CD27^−^ T-cells are protective effector cells is mainly based on the fact that these cells possess strong cytotoxic potential, expressing high levels of perforin, as seen ex vivo (Hamann et al. 1997). However, a recent report suggests that ex vivo Cr51 release assay, and therefore perforin levels, may not be a true reflection of in vivo cytotoxic capacities and, accordingly, this could be misleading in the interpretation of what constitutes a protective “effector cell” (Barber et al. 2003).

It was previously reported that antigen-specific CD27^−^ CD8^+^ T-cells do proliferate (van Leeuwen et al. 2002). We show here that only a proportion of highly differentiated CD27^−^ CD8^+^ T-cells express CD57, therefore exhibiting reduced proliferative capacities, while the rest of the CD27^−^ CD8^+^ T-cells should indeed be able to expand. Nonetheless, the vast majority of highly differentiated cells with high levels of perforin are CD57^+^ (Figure 5E). The association between high levels of perforin and characteristics of replicative senescence is not a particular characteristic of HIV infection, but holds true in both HIV-infected and HIV-noninfected individuals (Figure 5F). Increase in the intracellular perforin content seems to be the normal consequence of the process of post-thymic development, and it is also valid in the case of CD4^+^ T-cell differentiation, since cytotoxic CD4^+^ T-cells, whose proportions are increased during HIV-1 infection (Appay et al. 2002c), are CD57^+^ (Figure 5G). Overall, as HIV-1-infected individuals are progressing, they display increasing proportions of late-differentiated T-cells with characteristics of replicative senescence, with an average of 40% of CD57^+^ CD8^+^ T-cells in progressor/AIDS individuals (data not shown). Overall, the accumulation of highly differentiated CD8^+^ T-cells in HIV infection goes along with reports of reduced proliferative capacities and shorter telomere length characterising the T-cells of the HIV-infected individual (Wolthers et al. 1996a; Bestilny et al. 2000; Effros 2000).

## Discussion

Here we have studied the interplay between CD8^+^ T-cell activation and differentiation and its implications for HIV pathogenesis. HIV-1 induces a strong immune activation, which is particularly evident within the CD8^+^ T-cell compartment. Our data indicate that HIV-1 infection results in immune activation not only directly, but also indirectly, with the activation of cells specific for non-HIV antigens. In recent years, the role of potential bystander activation has been reevaluated and is now considered less important (Murali-Krishna et al. 1998), suggesting that most of the stimulation observed may be antigen-driven. During acute HIV-1 infection, immunosuppression may develop that favours the replication of host flora like CMV and EBV, as occurs in other immunocompromised individuals (Yao et al. 1996; Gerna et al. 1998). Recently, the help provided by CD4^+^ T-cells to control viral replication has been emphasised in the context of CMV infection (Gamadia et al. 2002). The drop in the CD4^+^ T-cell counts during HIV acute infection may result in suboptimal immune control of CMV and EBV and thus permits the replication of these viruses. Data have indicated that frequent reactivation of CMV likely occurs in the human host, as evidenced by the presence of a large population of CD69^+^ CMV-specific cells, indicative of recent in vivo activation (Dunn et al. 2002). Hence, HIV infection may serve to increase both the frequency and magnitude of CMV reactivation. In addition, inflammatory conditions occurring during HIV acute infection (e.g., release of proinflammatory cytokines) may participate in the reactivation of latent forms of CMV and EBV.

We have shown here that T-cell activation and increasing differentiation are closely related. One could speculate that the association between different stages of CD8^+^ T-cell differentiation and viral specificity of these cells, as previously described (Appay et al. 2002a; Tussey et al. 2003), may be related to the stimulation intensity received by the cells from priming onwards. CMV may therefore be a particularly potent stimulus for CD8^+^ T-cells, thus promoting a strong differentiation of these cells. Interestingly, a similar phenomenon seems to happen in the context of CD4^+^ T-cells, as CMV-specific CD4^+^ T-cells show further differentiation, in comparison with EBV-specific CD4^+^ T-cells (Amyes et al. 2003).

In the context of HIV infection, elevated and chronic immune activation is the most plausible cause for the general shift of the CD8^+^ T-cell population towards the highly differentiated cells that accompanies progression towards AIDS, as we have shown that elevated cellular activation drives further differentiation of CD8^+^ T-cells (including HIV-, CMV-, or EBV-specific cells). Converging evidence suggests that a reduction of replicative potential occurs with extensive T-cell division and differentiation. Differentiation towards late stages (CD28^−^/CD27^−^/CD57^+^) is strongly associated with the display of characteristics of replicative senescence, which may have an impact on viral control. The relevance of perforin^high^ late-differentiated T-cells in conferring protective immunity is controversial. For instance, van Baarle et al. (2002a) reported a correlation between high numbers of late-differentiated HIV-specific CD8^+^ T-cells and years of AIDS-free survival. However, it remains to be determined whether late-differentiated CD8^+^ T-cells would simply accumulate in these individuals with chronic infection over time, whilst playing no role in delaying disease progression. Overall, there is confusion regarding the ideal functional and phenotypic profile of a “protective effector cell.” Protective immunity has recently been associated with the proliferative capacity of virus-specific CD8 T-cells in the mouse model (Wherry et al. 2003). This is supported by Migueles et al. (2002), who showed that HIV-1-infected long-term nonprogressors are characterised by HIV-1-specific CD8^+^ T-cells that maintain a strong proliferative capacity following in vitro stimulation (cells defined mainly as CD45RO^+^/CD28^+^/CD27^+^ early-differentiated cells). In this study, the proliferative potential of these cells was coupled to strong perforin expression, suggesting that early-differentiated cells (which express low perforin levels in a resting state [Appay et al. 2002a]) are able to express high perforin levels after certain conditions of stimulation. In contrast, the high perforin levels observed in resting late-differentiated T-cells seem to correlate with characteristics of replicative senescence. These findings challenge the view that highly differentiated T-cells are beneficial effector cells that should be the goal of vaccine or immunotherapeutic strategies (Speiser et al. 2002). In keeping with this position, the fraction of perforin^high^ HIV-specific CD8^+^ T-cells has been proposed to be a marker for disease progression (Heintel et al. 2002). One may speculate that this high perforin expression may reflect an alteration of gene expression related to replicative senescence. This may not be dissimilar to the changes in gene expression that occur during replicative senescence in fibroblasts (Smith and Pereira-Smith 1996). More investigations on this matter will be necessary to clarify the cause and consequence of high perforin levels in late-differentiated T-cells.

The elevated and chronic stimulation induced by HIV-1 may result in the exhaustion of the capacity to generate new T-cells (Hazenberg et al. 2003), while the pool of antigen-experienced cells is driven to differentiate into aged oligoclonal populations. Interestingly, these characteristics are not unique to HIV infection, but they are also common to other conditions that result in some degree of immunodeficiency, like ataxia telangiectasia (Giovannetti et al. 2002), and normal human ageing (Nociari et al. 1999; Rufer et al. 1999). They may reflect a premature decline of the immune resources necessary for viral control and therefore contribute to the onset of disease progression (Effros 2000; Hazenberg et al. 2000a; Appay and Rowland-Jones 2002b; Grossman et al. 2002). This hypothesis is also strongly supported by a recent study performed in a mouse model in which persistent immune activation was shown to exhaust the T-cell pool and be sufficient to induce lethal immunodeficiency (Tesselaar et al. 2003). In addition to a direct effect of HIV on the thymus, decreased thymic output and T-cell renewal may originate from thymus involution (Kalayjian et al. 2003) as well as the failure of the bone marrow and the reduction of primitive hemaopoietic stem cell subsets (Marandin et al. 1996; Moses et al. 1998), as observed in HIV-1-infected individuals. Increased proportions of highly differentiated T-cells may relate to the maintenance of homeostasis and “immunological space” in the absence of T-cell renewal.

Our study also emphasises the importance of considering the influence of HIV-1 infection on other pathogens as well as the influence of these pathogens on HIV pathogenesis. For instance, CMV is known to drive substantial differentiation of T-cells towards CD57^+^ cells (Wang et al. 1995). CMV may therefore play an important role in the decline of the immune resources, as recently proposed in the HIV-noninfected elderly (Khan et al. 2002; Wikby et al. 2002). CMV infection was recently associated with a higher rate of disease progression in HIV-1-infected infants (Kovacs et al. 1999) and with reduced survival in patients with advanced HIV disease (Erice et al. 2003); it has also been shown to be a cofactor for HIV disease progression and death in some longitudinal studies of HIV-infected haemophiliacs (Webster et al. 1989). The impact of elevated activation and differentiation on immune function appears to have considerable importance in the onset of immunodeficiency and needs to be addressed in the development of current and future anti-HIV strategies.

## Materials and Methods

### 

#### Study subjects.

Samples were taken from HIV-1-infected patients attending clinics in London or Oxford (United Kingdom) and San Diego (United States) who were known to have either acute or chronic HIV-1 infection. The relevant local Institutional Review Boards and Ethics Committees approved the study. Subject ages ranged from 23 to 65 y old. Eleven patients with HIV-1 acute infection were selected from a well-characterised cohort in San Diego on the basis of their having an HLA type (HLA-A*0201, HLA-B*0701, or HLA-B*0801) for which we could detect virus-specific CD8^+^ T-cell populations using tetramers. The donors were diagnosed before or at the time of HIV-1 seroconversion, defined by symptomatic disease, recent high-risk exposure, high-plasma *HIV-1* RNA (ranging from 3 × 10^5^ to 3 × 10^6^ copies/ml [mean, 8.3 × 10^5^ copies/ml]), and either a negative HIV-1 ELISA or a negative/indeterminate HIV-1 Western blot. A second sample was analysed at a later timepoint after the start of successful ART (see Table 1). The study also involved untreated HIV chronically infected individuals: either with indications of viral control (*n* = 14, drug naïve, infected for more than 10 y with a CD4^+^ count above 500 per milliliter and viral load ranging from undetectable to 2 × 10^4^ copies/ml) or with evidence of progressive HIV disease (*n* = 10, with decreasing CD4^+^ count, 500 < *x* < 130 per milliliter, and viral load ranging from 5 × 10^3^ to 3 × 10^5^ copies/ml). Blood samples were also obtained from healthy adult volunteers. Peripheral blood mononuclear cells (PBMCs) were separated from heparinised blood and cryopreserved for subsequent studies. HLA typing was carried out by amplification refractory mutation system–polymerase chain reaction (ARMS–PCR) using sequence-specific primers as previously described (Bunce et al. 1995). HLA-typed patients were generally screened first for virus-specific CD8^+^ T-cell responses by means of Elispot assays using known HLA class I-restricted viral epitope peptides.

#### Reagents and flow cytometry.

HLA–peptide tetrameric complexes (“tetramers”) were produced as previously described (Altman et al. 1996) and included the following specificity: A2 HIV p17-SLYNTVATL and pol-ILKEPVHGV, A2 CMV pp65-NLVPMVATV, A2 EBV BMLF1-GLCTLVAML, A2 influenza matrix-GILGFVFTL, A2 melan-A-ELAGIGILTV, B7 HIV nef-TPGPGVRYPL and gp41-IPRRIRQGL, B7 CMV pp65-TPRVTGGGAM, B8 HIV nef-FLKEKGGL and p24-DIYKRWII, B8 EBV BZLF1-RAKFKQLL, B35 CMV pp65-VFPTKDVAL and B57 HIV p24-KAFSPEVIPMF. Anti-CD8–PerCP (peridinin chlorophyll protein) or APC CY7 (allophycocyanin cyanine 7), anti-CD27–PE (phycoerythrin) or APC, anti-CD28–FITC (fluorescein isothiocyanate), anti-CD38–APC, anti-CD45RA–FITC or ECD (PE–Texas red), anti-CD62L–APC, anti-Ki67–FITC, anti-CD69–FITC, anti-CCR7-purified, anti-granzyme A–FITC, and anti-perforin–PE antibodies were purchased from Becton-Dickinson PharMingen (San Diego, California, United States); anti-CD57–FITC or PE antibodies were from Beckman Coulter (San Diego, California, United States).

FACS stainings were performed as previously described (Appay and Rowland-Jones 2002a). In brief, titrated tetramers (PE-conjugated) were added to 150 μl of heparinised blood or PBMCs, followed by addition of a panel of titrated antibodies (FITC-, PerCP-, or APC-conjugated). The lymphocytes were then fixed and the red blood cells lysed using FACS^TM^ lysis solution (Becton-Dickinson). Cells were washed, fixed, and permeabilised in FACS^TM^ permeabilisation buffer (Becton-Dickinson). After washing, intracellular perforin staining was performed using titrated antibodies. Cells were then washed and stored in Cell Fix^TM^ buffer (Becton-Dickinson) at 4°C until analysis. Samples were analysed on a Becton-Dickinson FACSCalibur after compensation was checked using freshly stained PBMCs. Carboxyfluorescein diacetate succinimidyl ester (CFSE) labeling was performed by incubating PBMCs with 5 μM CFSE (Molecular Probes, Leiden, The Netherlands) in RPMI 1640 for 10 min at 37°C, before quenching with ice-cold RPMI 1640–10% foetal calf serum (FCS) and washing. The cells were then incubated with immobilised OKT3 (10 μg/ml) for 6 d (with or without 20U/ml of IL-2) before staining.

#### Flow fluorescence in situ hybridisation.

Naïve and antigen-experienced CD8^+^ T-cell subsets were sorted ex vivo from freshly isolated PBMCs, on the basis of CD27, CD57, CCR7, and CD45RA expression using a five-color FACS vantage SE (with 98%– 99% purity). For each subset, 0.5 × 10^5^ to 2 × 10^5^ cells were used to measure the average length of telomere repeats at chromosome ends in individual cells by quantitative flow fluorescence in situ hybridisation (FISH), as previously described (Rufer et al. 1998, 1999). FITC-labeled fluorescent calibration beads (Quantum TM-24 Premixed; Bangs Laboratories Inc., Fishers, Indiana, United States) were used to convert telomere fluorescence data to molecules of equivalent soluble fluorescence (MESF) units. The following equation was performed to estimate the telomere length in basepairs from telomere fluorescence in MESF units: basepair = MESF × 0.495 (Rufer et al. 1998).

#### In vitro priming of CD8^+^ T-cells with DCs.

DCs were generated as previously described (Salio et al. 2001). Monocytes were purified from healthy donors' PBMCs (screened for HLA-A2 expression) by positive sorting using anti-CD14-conjugated magnetic microbeads (Miltenyi Biotec, Bergisch-Gladbach, Germany). The recovered cells were greater than 99% CD14^+^. DCs were generated by culturing monocytes in RPMI 1640–10% FCS supplemented with 50 ng/ml GM–CSF (Leucomax, Basel, Switzerland) and 500 U/ml IL-4 (Peprotech, London, United Kingdom) for 5 d. Cells (3 × 10^5^/ml) were stimulated by addition of 1 μg/ml LPS (Sigma, St. Louis, Missouri, United States). Antigen-presenting cells were pulsed for 3 h with various concentrations of melan-A–peptide in serum-free medium before incubation with autologous PBMCs at a 1:5 ratio in RPMI 1640–10% FCS. Human rIL-2 (R&D Systems, Minneapolis, Minnesota, United States) was added from day 4 at 10 U/ml, then at 500 U/ml IL-2 when cells expanded. Melan-A-specific CD8^+^ T-cells were then analysed by flow cytometry over time for up to 50 d.

#### Statistics.

Group medians and distributions were compared by the nonparametric Mann–Whitney test. Associations between variables were determined by the nonparametric Spearman rank correlation test. Associations between variables in different patient groups were determined by simple linear regression or ANOVA test. *P* values above 0.05 were considered not significant.

## Abbreviations

AIDS: acquired immunodeficiency syndrome
APC: allophycocyanin
ARMS–PCR: amplification refractory mutation system–polymerase chain reaction
ART: antiretroviral therapy
CFSE: carboxyfluorescein diacetate succinimidyl ester
CMV: cytomegalovirus
CY7: cyanine 7
DC: dendritic cell
EBV: Epstein–Barr virus
FCS: foetal calf serum
FISH: fluorescence in situ hybridisation
FITC: fluorescein isothiocyanate
HIV: human immunodeficiency virus
HLA: human leukocyte antigen
IFN: interferon
IL: interleukin
MESF: molecules of equivalent soluble fluorescence
PBMC: peripheral blood mononuclear cell
PE: phycoerythrin
PerCP: peridinin chlorophyll protein
SIV: simian immunodeficiency virus
